# Neutrophil-lymphocyte ratio, red cell distribution width and mean platelet volume as practical markers in febrile seizure classification

**DOI:** 10.1590/1984-0462/2024/42/2023016

**Published:** 2023-11-03

**Authors:** Pelin Balikoğlu, Ayse Oflu, Ayşegül Bükülmez

**Affiliations:** a Afyonkarahisar Health Sciences University, Faculty of Medicine, Department of Pediatrics, Afyonkarahisar, Turkey.

**Keywords:** Febrile seizures, Neutrophil lymphocyte, Mean platelet volume, Convulsões febris, Neutrófilos linfócitos, Volume médio de plaquetas

## Abstract

**Objective:**

To examine the neutrophil-lymphocyte ratio, red cell distribution width and mean platelet volume in patients with febrile seizure and to determine their role in febrile seizure classification.

**Methods:**

This was a retrospective hospital-based study conducted among patients aged 5 to 72 months admitted with febrile seizure. Children who had febrile seizures due to upper respiratory tract infection were included in the study. The children were divided into two groups: simple febrile seizures and complex febrile seizures. Patients with a history of febrile status epilepticus, previous convulsions, use of antiepileptic or other chronic drugs, foci of infection other than the upper respiratory tract infection, abnormal biochemical parameters, and chronic mental or physical disease were excluded from the study. Clinical and laboratory findings of the patients were obtained from digital medical records.

**Results:**

The records of 112 febrile seizure patients were reviewed, and 89 were grouped as simple and 23 as complex febrile seizures. Although there was no statistically significant difference between the two groups in terms of the mean red cell distribution width values (p=0.703), neutrophil-lymphocyte ratio and mean platelet volume were significantly higher in patients with complex febrile seizures (p=0.034, p=0.037; respectively).

**Conclusions:**

This study showed that neutrophil-lymphocyte ratio and mean platelet volume could be practical and inexpensive clinical markers for febrile seizure classification. A similar result could not be reached for red cell distribution width in this study. These findings should be supported by multicenter studies with large samples.

## RESUMO

**Objetivo:**

Examinar a relação linfócitos-neutrófilos, amplitude de distribuição de hemácias e volume médio de plaquetas em pacientes com convulsão febril, e determinar seu papel na classificação de convulsão febril.

**Métodos:**

Este foi um estudo retrospectivo de base hospitalar realizado com pacientes de 5 a 72 meses admitidos com convulsão febril. Crianças que tiveram convulsões febris em razão de infecção do trato respiratório superior foram incluídas no estudo. As crianças foram divididas em dois grupos: convulsões febris simples e complexas. Pacientes com história de Status epiléptico febril, convulsões prévias, uso de drogas antiepilépticas ou outras drogas crônicas, com focos de infecção que não a do trato respiratório superior, parâmetros bioquímicos anormais e doenças crônicas mentais ou físicas foram excluídos do estudo. Os achados clínicos e laboratoriais dos pacientes foram obtidos a partir dos prontuários médicos digitais.

**Resultados:**

Registros de 112 pacientes com convulsão febril foram revisados: 89 com convulsões febris simples e 23 com complexas. Embora não tenha havido diferença estatisticamente significativa entre os dois grupos em termos de valor médio de amplitude de distribuição de hemácias (p=0,703), a relação linfócitos-neutrófilos e o volume médio de plaquetas foram significativamente mais elevados em pacientes com convulsões febris simples (p=0,034, p=0,037; respectivamente).

**Conclusões:**

Este estudo mostrou que a relação linfócitos-neutrófilos e o volume médio de plaquetas podem ser marcadores clínicos práticos e de baixo custo para a classificação de convulsão febril. Um resultado semelhante não pôde ser alcançado para a amplitude de distribuição de hemácias neste estudo. Esses achados devem ser apoiados por estudos multicêntricos com grandes amostras.

## INTRODUCTION

Febrile seizure (FS) is a type of convulsion typically seen in children between six months and six years of age that is not triggered by central nervous system infection or inflammation. It is divided into two groups — simple and complex — according to their clinical features. Simple febrile seizures (SFS) take less than 15 minutes, do not recur within 24 hours, and cause no cognitive impairment.^1^ Complex febrile seizures (CFS) are characterized by recurrences within 24 hours, accompanied by focal neurological findings, and last longer than 15 minutes. It is known that 25–30% of FS are complex.^2,3^ Most febrile seizures occur outside healthcare facilities, and information on the character of the seizures is usually obtained from the parents. There is no objective parameter in the current literature to distinguish between these two types of seizures. For this reason, objective diagnostic indicators are needed to determine them.

Currently, neutrophil-lymphocyte ratio (NLR) has gained importance as a hematological marker that can be used in the differential diagnosis of many diseases and in determining their severity and prognosis. The basis of previous studies investigating NLR as a practical inflammatory marker is the physiological response of leukocytes to inflammatory stimuli, an increase in neutrophil count, and a relative decrease in lymphocyte count accompanying neutrophilia. In intensive care practice, the ratio of these two leukocyte subgroups is used as an inflammation marker.^4^ NLR has been shown to be associated with increased inflammation in various systemic diseases.^5^ Few recent studies have also reported that NLR is a practical marker for distinguishing FS types.^6,7^

While NLR has emerged as a new marker for inflammation, red blood cell distribution width (RDW) and mean platelet volume (MPV) have also begun to be investigated as two additional practical markers for inflammation. Although the mechanism is not fully clear, RDW has been reported in some studies as a new indicator of inflammation in various diseases.^7^ As a parameter reflecting the rate of platelet production from the bone marrow, MPV is a machine-calculated measure of the mean platelet size. Today, besides being a parameter showing platelet activation, it has also been reported as another marker of inflammation severity.^8^

Considering that cell blood count (CBC) parameters are practical and inexpensive laboratory tests, revealing the roles of these markers in FS classification may be beneficial in emergency service practice. Although there are studies on this subject, both the limited number of papers and the low number of cases in the existing papers show that there is a need for new studies. Therefore, the present study aimed to examine NLR, RDW, and MPV in patients with FS and determine their role in FS classification.

## METHOD

This retrospective study was conducted in the pediatric clinic of a tertiary hospital between August 15, 2017 and February 15, 2018. The digital medical files of patients aged 5 to 72 months who applied with febrile seizure complaints between January 01, 2014 and December 31, 2017 and underwent routine CBC examination in the first 24 hours were evaluated retrospectively. A diagnosis of FS was determined according to the International Classification of Diseases, Ninth Revision (ICD-9; codes 780.31 and 780.32). The study was conducted based on the principles of the Declaration of Helsinki and approved by the local ethics committee on February 03, 2017, under number: 2017-2:39.

Children with fever due to upper respiratory tract infection and symptoms such as fever, sore throat, nasal congestion, tonsillitis, and pharyngitis, without a history of febrile seizures or who had seizures within the first three days after the onset of febrile illness were included in the study. The study group was divided into SFS and CFS. SFS was defined as a seizure that lasted less than 15 minutes, did not recur within 24 hours, was not accompanied by focal neurological findings, and had no central nervous system infection. CFS was defined as a seizure that lasted longer than 15 minutes, recurred within 24 hours, and was accompanied by focal neurological findings or postictal paresis. Patients with a history of febrile status epilepticus, previous convulsions, using antiepileptic or other chronic drugs, the focus of infection other than upper respiratory tract, abnormal biochemical parameters, and chronic mental or physical disease were excluded from the study.

With a retrospective analysis of laboratory parameters, white blood cell (WBC), hemoglobin (Hb), hematocrit (Hct), RDW, platelet count (PLT), mean corpuscular volume (MCV), MPV, neutrophil and lymphocyte counts, neutrophil and lymphocyte percentages, NLR, and C-reactive protein (CRP) level were examined. The NLR was obtained by dividing the number of neutrophils by the number of lymphocytes. These parameters were obtained from the patient's first laboratory examinations until the first two hours of admission.

Data were evaluated using descriptive statistics such as arithmetic mean±standard deviation, median and percentage distribution. The compliance of continuous data to normal distribution was evaluated through the Shapiro-Wilk test. When comparing the quantitative data of two independent groups, the independent group t-test was applied in cases where parametric conditions were met, otherwise, the Mann-Whitney U test was applied. While comparing the percentage distribution of categorical data between groups, the chi-square test was used. Receiver Operating Characteristic (ROC) curve analysis was used for calculating the optimal cutoff values, sensitivity, and specificity of NLR and MPV. Data analysis was performed using Statistical Package for Social Sciences (SPSS) version 20.0. Values of p<0.05 were considered statistically significant.

## RESULTS

The mean age of 112 patients with febrile convulsions included in the study was 19.7±7.2 months and 52 (46.4%) were female. Of the total, 89 (79.5%) were classified as SFS and 23 (20.5%) as CFS. There was no difference between the groups in terms of age (p=0.295) and gender (p=0.817).

The mean NLR was 2.06±1.99 in SFS and 3.30±3.81 in CFS. NLR value was found to be significantly higher in patients with CFS (p=0.034). The MPV value was 7.67±1.13 fL in SFS and 8.32±1.94 fL in CFS. MPV was found to be significantly higher in patients with CFS (p=0.037). RDW, other CBC parameters, and serum CRP values were compared, and no difference was observed between the two groups (p>0.05) (Table 1).

**Table 1:** Comparison of laboratory characteristics of patients with simple febrile seizure and complex febrile seizure.

	SFS group (n=89)	CFS group (n=23)	p
WBC (×103/mm^3^)*	12.8±6.9	14.3±6.1	0.357
Neutrophil count (×103/mm^3^)*	7.1±6.1	8.8 ±6.2	0.179
Lymphocytes count (×103/mm^3^)*	4.4±2.1	4.2±2.1	0.799
Neutrophil (%)*	49.0±21.3	54.8±20.9	0.249
Lymphocytes (%)*	40.3±20.3	34.4±18.7	0.211
NLR*	2.1±2.0	3.3±3.8	0.034
RDW(%)*	15.5±2.2	15.3±2.7	0.703
MCV(fl)*	74.4±6.7	72.0±15.3	0.260
PLT(x10^6^/mL)*	349±105.1	325±94.7	0.312
MPV(fl)*	7.6±1.1	8.3±1.9	0.037
CRP (mg/dl)*	2.0±2.8	3.0±3.1	0.153

SFS: simple febrile seizure, CFS: complex febrile seizure; p: p-value; WBC: white blood cell; NLR: neutrophil lymphocyte ratio; RDW: red blood cell distribution width; MCV: mean corpusculer volume; PLT: platelet count; MPV: mean platelet volume; CRP: C-reactive protein.

* Values are expressed as mean±standard deviation.

**Figure 1:**
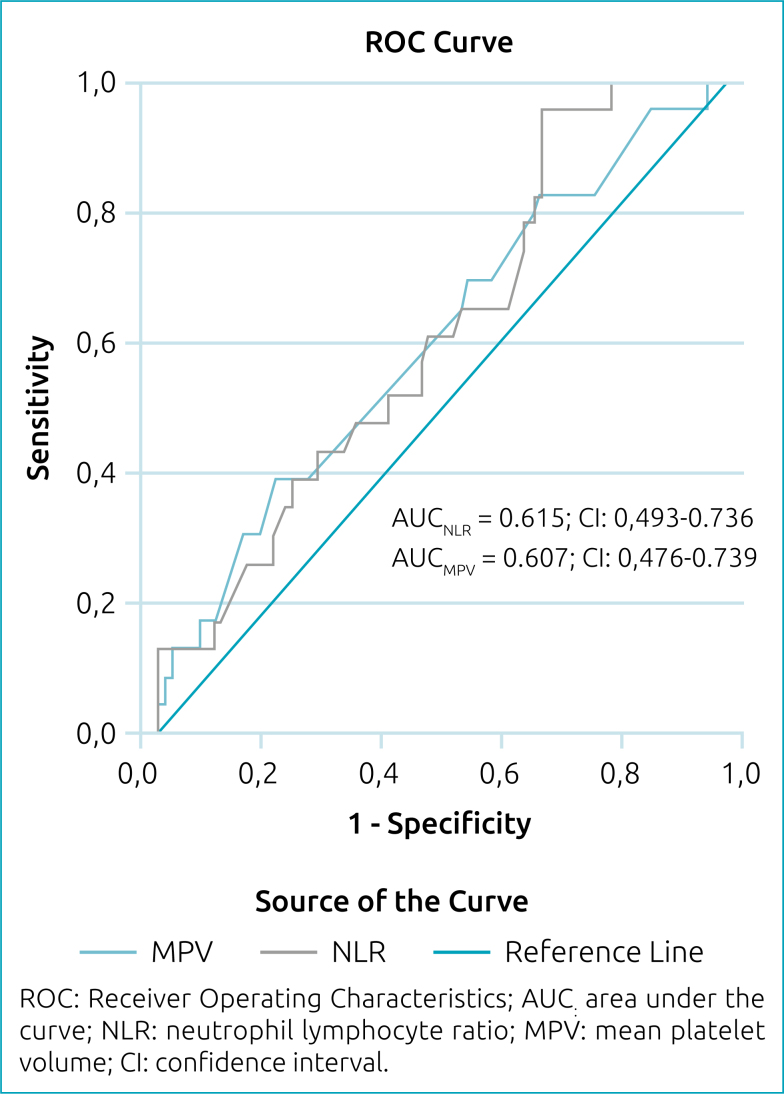
Receiver Operating Characteristics curve of neutrophil lymphocyte ratio and mean platelet volume for discriminating simple febrile seizure from complex febrile seizure.

According to ROC curve analysis in differentiating between SFS and CFS, the optimal cutoff value for NLR was found to be 1.59 (56.5% sensitivity; 53.9% specificity; area under the curve (AUC) 0.615; confidence interval [CI] 0.493–0.736) and for MPV, it was found to be 7.65 (56.5% sensitivity; 56.2% specificity; AUC 0.607; CI 0.476–0.739) (Figure 1).

## DISCUSSION

The present study showed that NLR and MPV values were significantly higher in patients with CFS than with SFS. In order to better understand this result, firstly, it is necessary to look at the relationship between FS and inflammation. Studies attempting to explain FS pathogenesis have shown that there is indeed a relationship between FS and inflammation. The effect of physiopathological processes that cause fever cannot be ignored in the development of FS — a problem that occurs during high fever or during the rapid rise of fever. It has been reported that interleukin-1 beta (IL-1β) and tumor necrosis factor-alpha (TNF-α), which are endogenous pyrogens involved in fever, play a role in the development of FS.^9-12^ It has been shown that IL-1β and TNF-α have direct and indirect modulating effects on neurons during inflammation.^13^ In previous studies, it was found that the production of IL-1β from peripheral blood monocytes was greater in patients with FS than in the control group.^14,15^ In addition, cortisol levels that increase with the stimulation of IL-1β may also cause leukocytosis, neutrophilia, and lymphopenia.^16,17^ It was also found that IL-1β infusion into the intracerebroventricular area increased neutrophil count and decreased lymphocyte count in peripheral blood.^18^

The NLR has been frequently investigated as a marker of systemic inflammatory status and a new parameter reflecting the systemic inflammation severity of various diseases.^19-21^ After revealing the relationship between inflammation and FS, recent studies have examined the NLR levels in patients with FS, and it has been shown to be significantly higher in children with FS compared to those without seizures.^7,22^ The relationship between the increase of neutrophils and the risk of FS was attributed to the effect of cytokines such as IL-1β and TNF-α released from neutrophils.^9,23^ It has also been reported that reactive oxygen radicals produced by neutrophils during inflammation are associated with the development of FS.^24,25^ Since it is a practical marker, the role of NLR in distinguishing FC types has also been investigated with few recent studies. They found that the NLR levels were significantly higher in the CFS group compared to the SFS.^6-8^ and the results of our study corroborated this finding. Vezzani et al.^26^ showed that IL-1β lowered the seizure threshold and worsened seizure activity. This finding also supports the relationship between CFS and higher NLR.

According to the ROC curve analysis in differentiating between SFS and CFS, the optimal cutoff value for NLR was 1.59. The sensitivity and specificity were 56.5% and 53.9%, respectively. Göksuğur et al.^6^ also found this limit value at 1.98 with sensitivity and specificity at 66.7% and 60.3%, respectively. Differences in cutoff value for NLR in both the present and previous studies may be related to the sample size of the studies.^6-8^

RDW is an index commonly used to investigate the etiology of anemia and describes the size variation of red blood cells. It has also been reported in previous studies that RDW may be an inflammatory marker, as it correlates positively with inflammatory markers such as erythrocyte sedimentation rate, CRP, and inflammatory cytokines.^27,28^ Results from previous studies regarding the relationship between FS types and RDW differed. Göksuğur et al.^6^ found that RDW was significantly higher in the CFS group compared to the SFS, but other studies showed that RDW was similar in both groups.^7,8^ The present study also showed that there was no difference between the two FS groups in terms of RDW.

Platelet activation has been reported as a common phenomenon in some diseases, and it has been suggested that inhibition of platelet activation may improve inflammation.^29^ PLT and MPV are used to evaluate platelet activation. High MPV is an indicator of larger, more reactive platelets resulting from increased platelet turnover and can be used as an indicator of platelet activation and inflammation severity.^30^ Although the role of MPV in distinguishing FS types has been investigated previously, the results obtained are not consistent. Some recent studies found an MPV significantly higher in the CFS group than in the SFS, while some studies showed no difference between the two groups. This study found that the MPV value was significantly higher in the CFS group. Conflicting results regarding the relationship between MPV, RDW, and FS types indicate that this relationship should be investigated in future studies with larger sample sizes.

The first limitation of this study is its small sample size. The second limitation is the unknown effect of a wide variety of upper respiratory tract infections on inflammation severity. However, the fact that the CRP value did not differ between the two groups suggests that this effect can be ignored. The third limitation is that although NLR and MPV have been identified as biomarkers of CFS in the context of acute inflammation in both previous and the present study, the pathophysiology of this relationship needs to be clarified. On the other hand, a strength of our study is that it contributes to the literature with its findings due to the limited number of studies conducted on this subject.

The present study demonstrated that NLR and MPV were both increased in CFS patients compared to SFS patients. This study also showed that the RDW value did not change in both groups. We think that NLR as a practical marker for clinicians will be beneficial in decision-making, such as observing patients with high NLR for at least 24 hours. Therefore, multicenter studies with larger samples are needed to determine more accurate cutoff values for NLR. Although NLR seemed to be a practical indicator that enables the differentiation of FS types due to the similarity of the findings in the studies carried out so far, further studies are needed to determine the role of MPV and RDW in this distinction due to the different results of previous studies.
